# Establishment of a prognostic model toward lung squamous cell carcinoma based on m^7^G-related genes in the cancer genome atlas

**DOI:** 10.1152/physiolgenomics.00149.2022

**Published:** 2023-08-14

**Authors:** Yongheng Wang, Yimin Liu, Rui Wang, Fuyuan Cao, Yi Guan, Yulu Chen, Binbin An, Sisi Qin, Sanqiao Yao

**Affiliations:** ^1^School of Public Health, North China University of Science and Technology, Tangshan, People’s Republic of China; ^2^School of Public Health, Xinxiang Medical University, Xinxiang, People’s Republic of China

**Keywords:** bioinformatics, lung squamous cell carcinoma, molecular docking, 7-methylguanosine modification

## Abstract

Lung squamous cell carcinoma (LUSC) is a non-small cell lung cancer with a poor prognosis owing to late diagnosis. New molecular markers are urgently needed to improve the diagnosis and prognosis of LUSC. 7-Methylguanosine (m^7^G) modifications, a tRNA modification, are common in eubacteria, eukaryotes, and a few archaea. These modifications promote the turnover and stability of some mRNAs to prevent mRNA decay, improve translation efficiency, and reduce ribosomal pausing but are associated with poor survival in human cancer cells. However, expression of m^7^G-related genes in LUSC and their association with prognosis remain unclear. In the present study, we identified nine differentially expressed genes related to prognosis by comparing the expression profiles of tumor tissues (502 LUSC reports) with normal tissues (49 adjacent nontumor lung tissue reports). The genes included six upregulated genes (*KLK7*, *LCE3E*, *AREG*, *KLK6*, *ZBED2*, and *MAPK4*) and three downregulated genes (*ADH1C*, *NTS*, and *ERLIN2*). Based on these nine genes, patients with LUSC were classified into low- and high-risk groups to analyze the trends in prognosis. We found that the nine m^7^G-related genes play important roles in immune regulation, hormone regulation, and drug sensitivity through pathways including antigen processing and presentation, adherent plaques, extracellular matrix receptor interactions, drug metabolism of cytochrome *P*-450, and metabolism of cytochrome *P*-450 to xenobiotics; the functions of these genes are likely accomplished in part by m^6^A modifications. The effect of m^7^G-related genes on the diagnosis and prognosis of LUSC was further indicated by population analysis.

**NEW & NOTEWORTHY** Based on the differential expression of 7-methylguanosine (m^7^G) modification-associated genes between normal and lung squamous cell carcinoma (LUSC) tissues, and considering the performance of our m^7^G-related gene risk profiles as independent risk factors in predicting overall survival, we conclude that m^7^G modification is closely linked to the development of LUSC. In addition, this study offers a new genetic marker for predicting the prognosis of patients with LUSC and presents a crucial theoretical foundation for future investigations on the relationship between m^7^G modification-related genes, immunity, and drug sensitivity in LUSC.

## Graphical abstract

**Figure:**
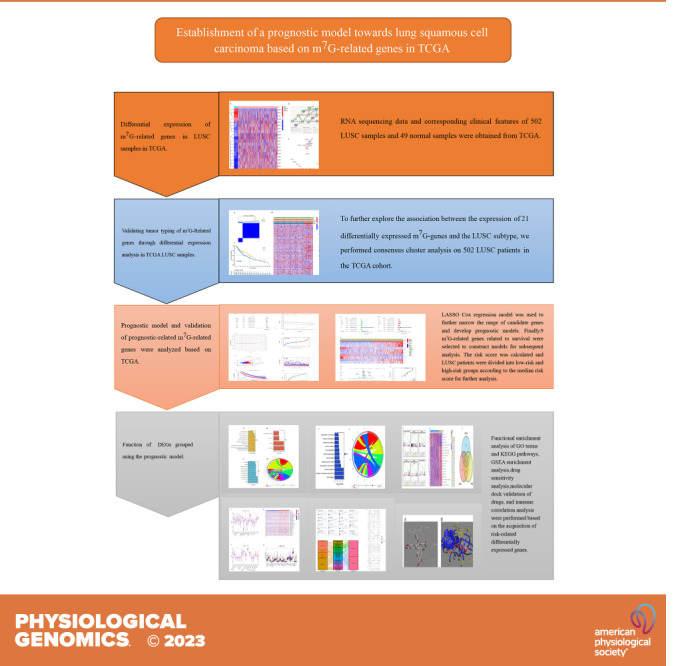

## INTRODUCTION

Lung cancer, one of the deadliest malignancies, has two main subtypes: non-small cell lung cancer (NSCLC) and small cell lung cancer (SCLC). NSCLC accounts for ∼85% of lung cancer cases, which can be classified into lung adenocarcinoma (LUAD) and lung squamous cell carcinoma (LUSC) based on pathogenesis and tumor histology (1–4). LUSC accounts for ∼40% of NSCLC cases and is characterized by poor prognosis and high rates of recurrence and metastasis. Many therapeutic approaches are currently applied for lung cancer, including conventional oncological therapies such as surgical resection, chemotherapy, radiotherapy, targeted therapy, and immunotherapy. NSCLC, including LUSC, can be surgically resected in the early stages of tumorigenesis, whereas advanced NSCLC is not amenable to resection. As NSCLCs are most often diagnosed in their advanced stages, a combinatorial targeted, immunochemotherapy approach is considered the most promising (5–9). Nevertheless, identifying biomarkers for LUSC is of considerable importance for the development of effective diagnostic, prognostic, and treatment strategies (10, 11).

tRNA, a classical noncoding RNA, can provide amino acids to ribosomes by forming a precise L-shaped structure based on the mRNA codon as an articulated molecule, a function that requires tRNA modifications (12, 13). To date, nearly 100 modified nucleosides have been reported in different RNA families (14–16). tRNA modifications are the most widespread among over 160 mRNA nucleotide variants (16, 17), among which methylation is the most prevalent modification (15, 18, 19). Recent studies have shown that 7-methylguanosine (m^7^G) may be associated with tumorigenesis. As a conserved modified nucleoside, m^7^G is primarily found in eubacteria and eukaryotes (17, 19, 20). m^7^G contains pre-tRNA molecular structures and is thus thought to be generated immediately after transcription (21, 22). In tRNA, m^7^G has been reported to contribute to pathogenic infectivity and has been associated with various diseases. m^7^G modification can also promote the turnover and stability of some mRNAs to prevent decay, improve translation efficiency, and reduce ribosomal pausing, which is correlated with poor survival in human cancer cells (23, 24). However, no m^7^G modification has been reported in LUSC.

In the present study, we characterized the relationship between m^7^G-related genes and LUSC using The Cancer Genome Atlas (TCGA) data. The prognostic value and functions of m^7^G-related genes in LUSC were explored using bioinformatic techniques to elucidate potential markers for the diagnosis, prognosis, and treatment of LUSC.

## MATERIALS AND METHODS

### Data Collection and Collation

RNA sequencing and the corresponding clinical data were obtained from TCGA database (https://portal.gdc.cancer.gov/repository) using the Illumina HiSeq platform for a data set consisting of 502 LUSC samples and 49 normal samples. Data were normalized and corrected as previously described (25), with a threshold of |log_2_FC| > 1.0 (where FC is fold change) and an adjusted *P* value of <0.05 while excluding genes with a mean expression of <0.5 to ensure the screening of significantly expressed genes. The screening process was performed using the R package “limma.”

### Identification of m^7^G-Related Genes

We identified 29 m^7^G-related genes from the literature and gene set enrichment analysis (GSEA) website (http://www.gsea-msigdb.org/gsea/index.jsp): GOMF_RNA_CAP_BINDING, GOMF_RNA_7_METHYLGUANOSINE_CAP_BINDING, and GOMF_M7G_5_PPPN_DIPHOSPHATASE_ACTIVITY.

### Analysis of Differential Expression

The expression of m^7^G-related genes in LUSC and normal samples was derived from TCGA database, literature, and GSEA (http://gsea-msigdb.org/gsea/index.jsp). The differential expression of genes between tumor tissues and normal tissues was assessed using the R package “limma” (|log_2_FC| > 2, adjusted *P* value < 0.05). Based on these results, we constructed a heatmap of differential expression, protein interaction network (https://string-db.org/), and coexpression network. The m^7^G-related differentially expressed genes (DEGs) were classified as LUSC and normal samples in TCGA database.

### Establishment and Validation of the Prognostic Model

Univariate Cox regression analysis was applied to assess the prognostic value of m^7^G-related DEGs by estimating the correlation between each DEG and survival status in TCGA cohort. A consensus cluster analysis was performed to identify prognostically relevant m^7^G-related DEGs. Using the minor absolute construction and selection operator (LASSO) Cox regression model (based on the optimal λ value) to select the strongest performing candidate genes, we identified nine m^7^G-related DEGs for use in a prognostic model. A risk score was calculated by correcting TCGA expression data using the following formula: risk score = ∑7*_i_X_i_* × *Y_i_* (where *X* is a coefficient and *Y* is the gene expression level). Patients with LUSC in TCGA were divided into low- and high-risk groups according to the median risk score for further analysis. Kaplan–Meier analysis was used to compare survival rates between the two groups, followed by receiver operating characteristic (ROC) validation and principal component analysis (PCA).

### Independent Prognostic Analysis of Risk Scores

Independent prognostic analysis of the variables in the prognostic model was performed by univariate and multivariate Cox regression based on the corresponding clinical information for patients in TCGA cohort.

### Functional Analysis of DEGs Between Subgroups Based on Prognostic Models

The prognostic model was constructed based on the m^7^G-related DEGs, and 34 risk-related DEGs were screened between the high- and low-risk groups (|log_2_FC| ≥ 1 and false discovery rate < 0.05). Based on the risk-related DEGs, we performed a functional enrichment analysis of gene ontology (GO) terms and Kyoto encyclopedia of genes and genomes (KEGG) pathways, GSEA, gene set variation analysis (GSVA), drug sensitivity analysis, molecular docking validation of drugs, and immune correlation analysis (immune cell infiltration, immune function correlation, and immune checkpoint correlation analyses).

### Statistical Analysis

To compare the differences between normal and LUSC tissues, we used one-way ANOVA to compare gene expression levels; Pearson’s χ^2^ test to compare categorical variables, and the Kaplan–Meier curve, LASSO regression, and bilateral log-rank tests to compare survival rates. We used univariate and multivariate Cox regression models to assess the independent prognostic value of the risk models. The Mann–Whitney *U* test was used to assess differences in immune cell infiltration, immune function activation, and immune checkpoints between the high- and low-risk groups. All statistical analyses were performed using R software v4.1.2 (The R Foundation, Vienna, Austria).

## RESULTS

### Differential Expression of m^7^G-Related Genes in LUSC Samples in TCGA

**Figure 1.**
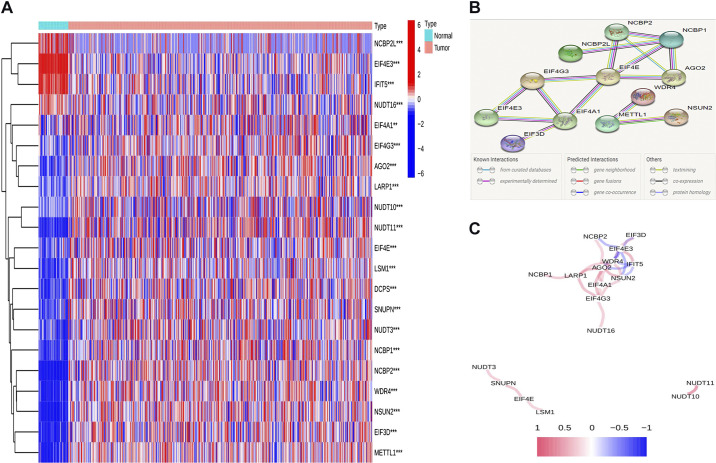
Expression and interaction of 7-methylguanosine (m^7^G)-related differentially expressed genes (DEGs). *A*: heatmap of m^7^G-related DEGs between normal (N; bright blue) and tumor tissues (T; red) (blue: low expression level and red: high expression level). **P < 0.01; ***P < 0.001. *B*: protein-protein interaction network of m^7^G-related DEGs (highest confidence level = 0.9). *C*: correlation network of m^7^G-related DEGs (red line: positive correlation and blue line: negative correlation; the depth of the color reflects the strength of the correlation).

We identified 21 m^7^G-related DEGs (all *P* < 0.01; Supplemental Table S1). The expression levels of these genes in LUSC versus normal samples are presented as a heatmap in Fig. 1*A.* Protein-protein interaction (PPI) analysis was performed on the STRING platform (https://cn.string-db.org/cgi/input?sessionId=bhF32mi3Zjuk) to assess the interaction of these m^7^G-related DEGs (Fig. 1*B*). The minimum interaction score for the PPI analysis was set at 0.9 (highest confidence level), and 12 genes were screened as core genes (*NCBP2*, *NCBP1*, *AGO2*, *NCBP2L*, *EIF4E*, *WDR4*, *EIF4G3*, *EIF4A1*, *EIF4E3*, *EIF3D*, *METTL1*, and *NSUN2*). The correlation network of the m^7^G-related DEGs is shown in Fig. 1*C.*

### Validation of Tumor Typing of m^7^G-Related Genes Based on Differential Expression of LUSC Samples in TCGA

**Figure 2.**
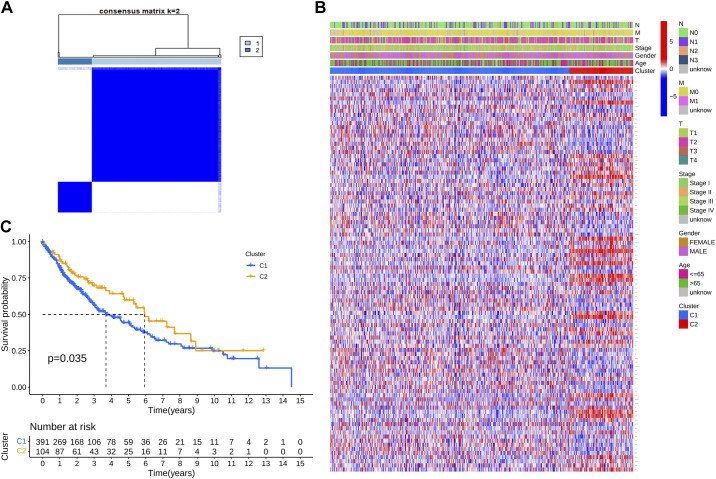
Lung squamous cell carcinoma (LUSC) cluster classification based on 7-methylguanosine-related differentially expressed genes (DEGs). *A*: patients with LUSC were divided into two clusters according to the consensus clustering matrix (*k* = 2). *B*: heatmap of the clinicopathological characteristics of the two clusters classified according to the DEGs. *C*: Kaplan–Meier overall survival curves for the two clusters.

To further explore the relationship between the expression of the 21 m^7^G-related DEGs and LUSC subtypes, we conducted a consensus cluster analysis of the 502 patients with LUSC. We set the clustering variable (*k*) to 2, given that the intragroup correlation was the highest and the intergroup correlation was low (Fig. 2*A*). The two clusters were further analyzed according to gene expression profiles and clinical features, including TNM stage (stage I–IV), age (≤65 or >65 yr), and survival status (survival or death; Fig. 2*B*). We found that the two clusters differed in their overall survival (OS) rates (*P* = 0.035; Fig. 2*C*).

### Construction and Validation of a Prognostic Model Based on m^7^G-Related DEGs

**Figure 3.**
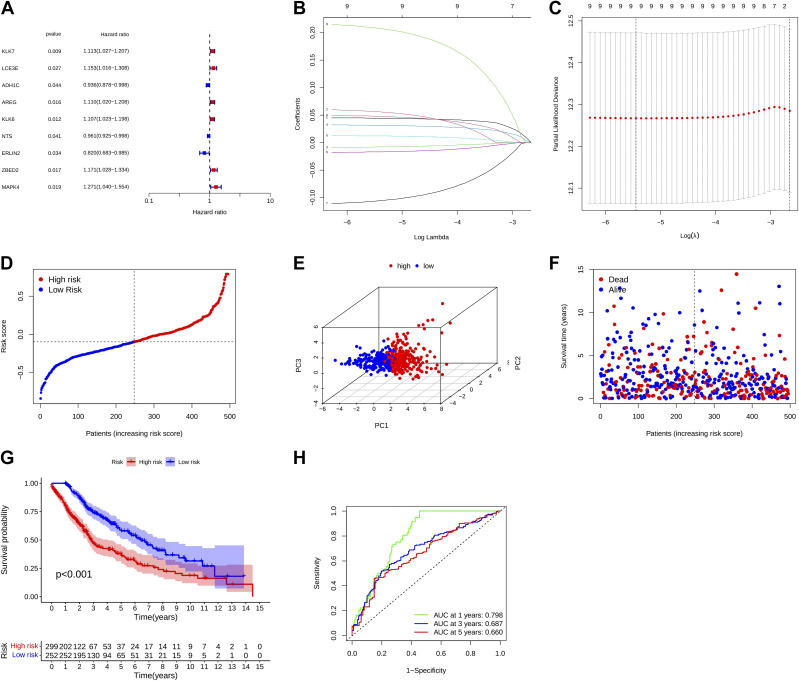
Construction and validation of prognostic model based on 7-methylguanosine-related differentially expressed genes. *A*: univariate Cox regression analysis of overall survival (OS) for each pyroptosis-related gene and seven genes with *P* < 0.05. *B*: LASSO regression of the seven OS-related genes. *C*: cross-validation for parameter selection in the LASSO regression. *D*: distribution of patients based on risk score. *E*: principal component analysis for OS based on the risk score. *F*: survival status (low-risk population: left of the dotted line and high-risk population: right of the dotted line). *G*: Kaplan–Meier OS curves of the high- and low-risk groups. *H*: receiver operating characteristic curves demonstrating the predictive efficiency of the risk score. AUC, area under the curve.

Of the nine prognostically relevant m^7^G-related DEGs identified by LASSO Cox regression, six genes (*KLK7*, *LCE3E*, *AREG*, *KLK6*, *ZBED2*, and *MAPK4*) had hazard ratios (HRs) of >1, suggesting that they were high-risk genes. The other three genes (*ADH1C*, *NTS*, and *ERLIN2*) had HRs of <1, suggesting that they were protective genes (Fig. 3, *A–C*). Risk scores were calculated as follows: risk score = (0.044 × *KLK7* exp.) + (0.056 × *LCE3E* exp.) + (–0.008 × *ADH1C* exp.) + (0.031 × *AREG* × exp.) + (0.012 × *KLK6* exp.) + (−0.017 × NTS exp.) + (−0.106 × *ERLIN2* exp.) + (0.048 × *ZBED2* exp.) + (0.208 × *MAPK4* exp.). Based on the median score, 252 and 299 patients were assigned to the low- and high-risk group, respectively (Fig. 3*D*). PCA showed a distinct distribution for patients with different risk scores (Fig. 3*E*). Patients in the high-risk group had higher mortality rates and lower OS rates (*P* < 0.001) than those in the low-risk group (Fig. 3, *F* and *G*). To further assess the sensitivity and specificity of the prognostic model, we performed a time-dependent ROC analysis. The areas under the curve (AUCs) of 0.798 at 1 year, 0.687 at 2 years, 0.660 at 3 years, and survival rates varied significantly between the risk groups (AUC > 0.650; Fig. 3*H*).

### Independent Prediction of Prognostic Gene Models

**Figure 4.**
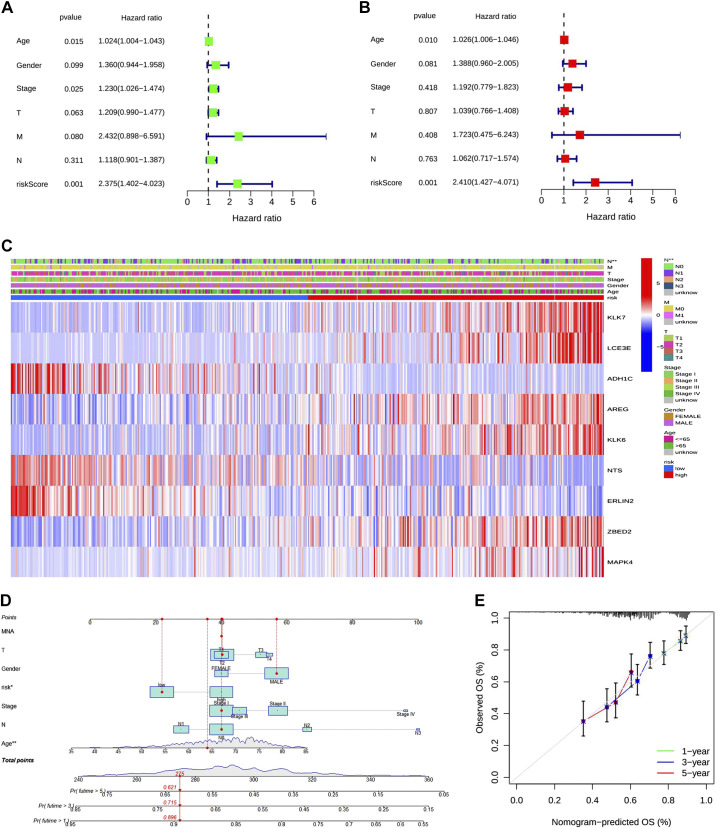
Univariate and multivariate Cox regression analysis. *A*: univariate analysis of The Cancer Genome Atlas (TCGA) cohort (grade: degree of tumor differentiation, G1−G3). *B*: multivariate analysis of TCGA queue. *C*: heatmaps of the association between clinicopathological features and risk group (blue: low expression and red: high expression). *P < 0.05. *D*: Rosette diagram of risk scores and outcomes. *E*: calibration curve. OS, overall survival *P < 0.05; **P < 0.01; ***P < 0.001.

Cox regression analysis was performed to assess whether the risk score of the prognostic model constructed from survival-related m^7^G-related DEGs could be used as an independent prognostic factor. Univariate Cox regression analysis showed that risk score, TNM stage, and age were independent factors relating to poor survival (*P* < 0.05; Fig. 4*A*). Further multivariate analysis showed that, after adjusting for other confounders, risk score and age were independent factors relating to poor survival (*P* = 0.010, HR = 1.026, and *P* = 0.001, HR = 2.410, respectively; Fig. 4*B*). A heatmap presenting the clinical characteristics of TCGA cohort is shown in Fig. 4*C.* To better demonstrate the relationship between risk scores and survival-related DEGs, we used column-line plots and calibration curves (Fig. 4, *D* and *E*) and found that the 1-, 3-, and 5-years survival curves were comparable with the standard curve, indicating a strong relationship between the risk score and survival prognosis in the prognostic model.

### Function of DEGs Grouped Using the Prognostic Model

The “limma” R package was used to analyze the grouping under the prognostic model, and DEGs were screened based on a false discovery rate < 0.05 and |log_2_FC| ≥ 1. We identified 34 DEGs between the low- and high-risk groups, among which 19 genes (*NTS*, *GSTA1*, *ADH1C*, *KRT13*, *KRT15*, *ADH7*, *SOX2*, *DAPL1*, *ALDH1A1*, *FOXA1*, *AKR1C2*, *GPC3*, *UPK1B*, *GPX2*, *CYP2S1*, *IGFBP2*, *LINC01133*, *ALOX15*, and *GSTA8P*) were downregulated and 15 (*PMEPA1*, *ZBED2*, *TGFBI*, *CDA*, *PI3*, *SBSN*, *CRCT1*, *KRT14*, *LCE3D*, *CST6*, *KLK5*, *AREG*, *KLK7*, *KLK6*, and *S100A7*) were upregulated. We performed functional analyses based on the 34 DEGs (Supplemental Table S2).

### GO Enrichment Analysis

**Figure 5.**
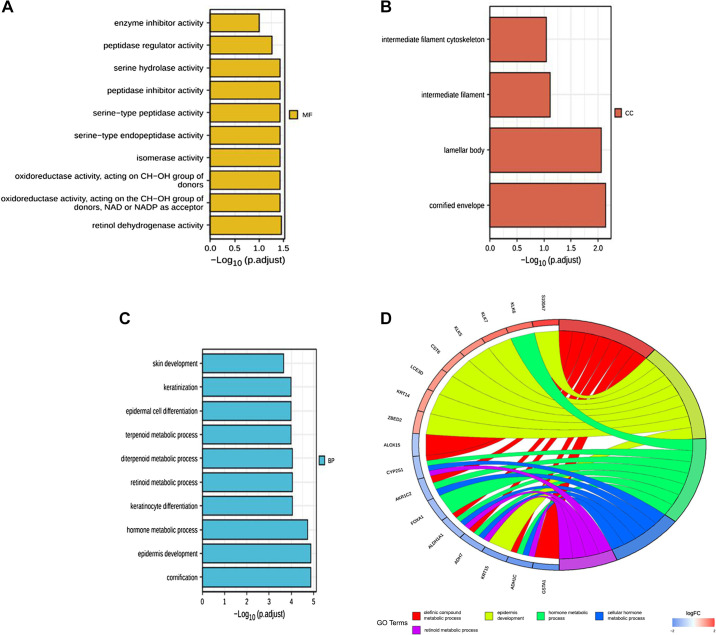
Gene ontology (GO) enrichment analysis of differentially expressed genes in the two risk groups. *A−C*: bar graphs of GO enrichment (longer bars indicate greater enrichment). *D*: circus diagram of GO results.

The GO enrichment analysis showed that the 34 DEGs were mainly related to the metabolic processes of olefin compounds, epidermal development, hormone metabolism, and vitamin A metabolism (Fig. 5).

### KEGG Functional Pathway Analysis

**Figure 6.**
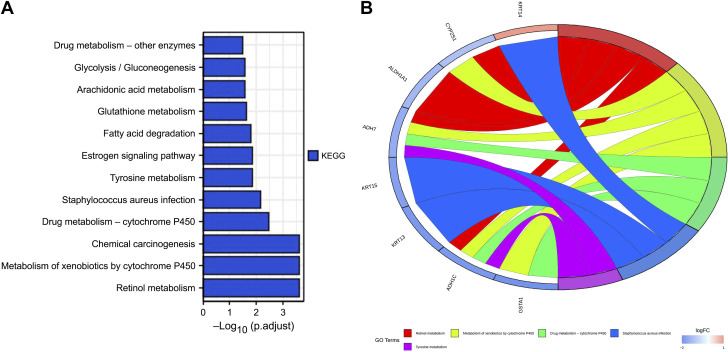
Kyoto encyclopedia of genes and genomes (KEGG) functional pathway analysis based on differentially expressed genes in the two risk groups. *A*: bar diagram of KEGG pathway enrichment (longer bars denote greater enrichment). *B*: circus diagram of KEGG results.

The KEGG pathway analysis showed that the 34 DEGs were mainly associated with retinol metabolism, cytochrome *P*-450 metabolism of xenobiotics, drug metabolism of cytochrome *P*-450, *Staphylococcus aureus* infection, and tyrosine metabolism (Fig. 6).

### GSVA Combined With GSEA

**Figure 7.**
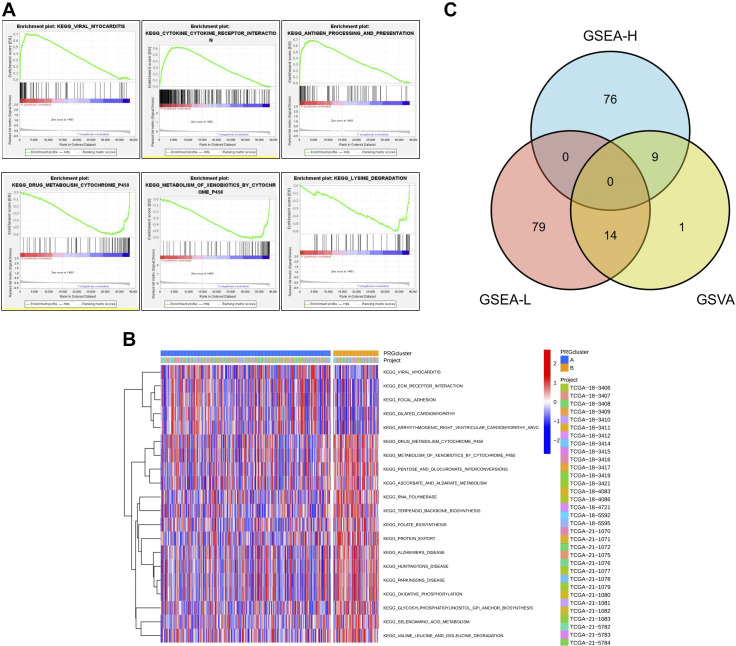
Gene set variation analysis (GSVA) and gene set enrichment analysis (GSEA) of differentially expressed genes. *A*: low and high expression pathways were obtained by GSEA. *B*: GSVA enrichment heatmap. *C*: intersection diagram of high and low expression pathways of GSEA and GSVA results.

To further characterize the function of the DEGs, 23 differential pathways were obtained by GSVA, including 9 high-expression pathways and 14 low-expression pathways (Fig. 7 and Supplemental Table S3). The top five functional pathways were antigen processing and presentation, adherent spot, extracellular matrix receptor interaction, drug metabolism of cytochrome *P*-450, and metabolism of cytochrome *P*-450 to xenobiotics.

### Immunological Correlation Analysis

**Figure 8.**
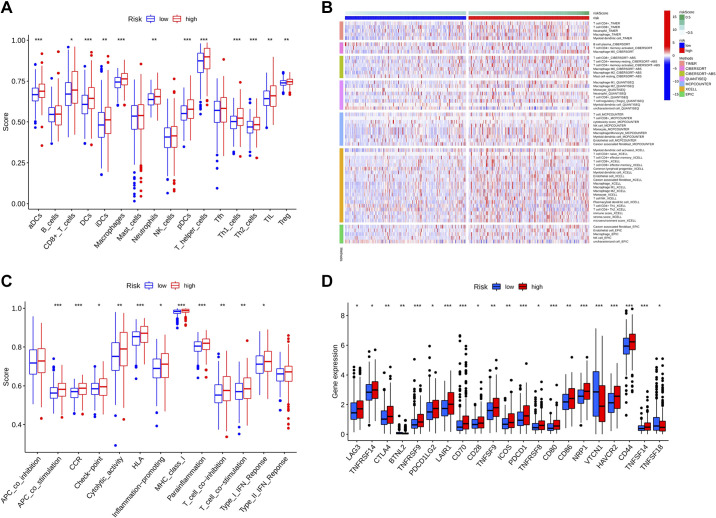
Immunological correlation analysis. *A*: immunocyte infiltration. *B*: analysis of immune cell correlation between high- and low-risk groups on different platforms. *C*: immune pathways. *D*: immune checkpoints. *P < 0.05; **P < 0.01; ***P < 0.001.

We performed an immune correlation analysis based on single-sample GSEA (ssGSEA) of 16 immune cell infiltrates, 13 immune-related pathways, and 21 immune checkpoints between the high- and low-risk groups. The analysis of immune cell infiltration, combined with the results of different platform simulations (Fig. 8, *A* and *B*), showed that the expression of immune cells was higher in the high-risk group than in the low-risk group except for B cells, mast cells, natural killer cells, and T follicular helper cells. Among these, “T helper cells” had a high rating and variability. Similarly, the expression of immune-related pathways was higher in the high-risk group than in the low-risk group except for antigen-presenting cell coinhibition and the type II interferon response. Particularly for “major histocompatibility complex class I,” the scores and variability were higher than in the other groups (Fig. 8*C*). In the analysis of immune checkpoints, all except “BTNL2,” “VTCN1,” and “TNFSF18” were highly expressed in the high-risk group. CD44 expression was also higher in the high-risk group than that in the low-risk group (Fig. 8*D*).

### Drug Sensitivity Analysis

**Figure 9.**
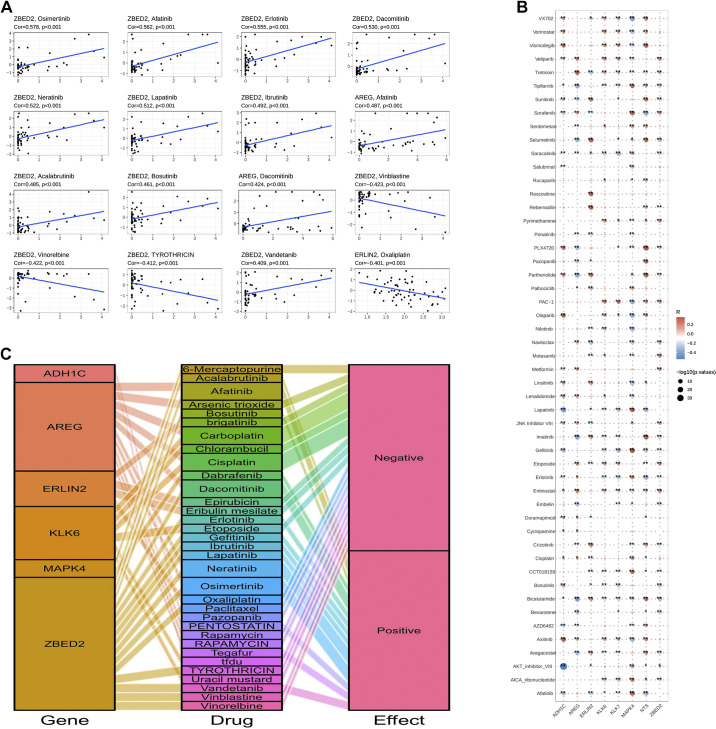
Drug sensitivity analysis. *A*: sensitivity analysis of the top 16 genes and drugs. *B*: drug sensitivity analysis matrix diagram. *C*: drug sensitivity analysis mulberry diagram. *P < 0.05; **P < 0.01; ***P < 0.001.

Nine m^7^G-related genes associated with prognosis were analyzed with drug sensitivity and transcriptomic data from the Genomics and Pharmacology Facility (https://discover.nci.nih.gov/) to obtain the final drug sensitivity analysis of the six m^7^G-related genes associated with prognosis. The top 16 pairs of genes were selected for scatterplot analysis against the corresponding drugs (Fig. 9*A*). Correlations of the six high-risk m^7^G-related genes and the corresponding drugs were analyzed by matrix and mulberry plots (Fig. 9, *B* and *C*).

### Molecular Docking Validation of the Drug Sensitivity Analysis

**Figure 10.**
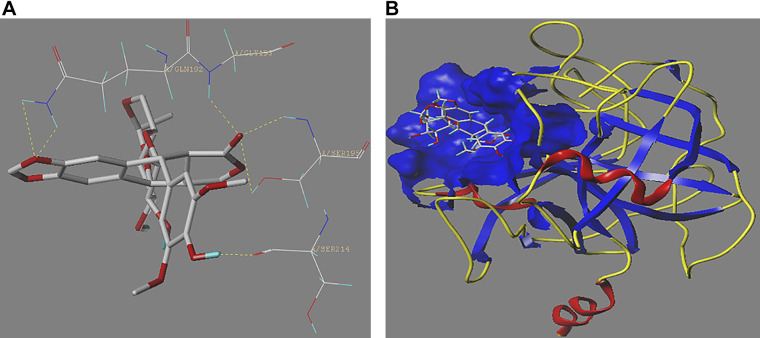
Molecular docking validation. *A*: three-dimensional structure of KLK6 and hydrogen bonding. *B*: docking model.

To further validate the results of the drug sensitivity analysis of the m^7^G-related DEGs, KLK6 was selected for molecular docking with four drugs, i.e., trifluridine, etoposide, cisplatin, and chlorambucil (Fig. 10 and Supplemental Table S4).

## DISCUSSION

In this study, we analyzed the expression of the 29 currently known m^7^G-related genes in LUSC against normal tissues based on TCGA data and identified 21 DEGs related to the prognosis. Consensus clustering analysis showed that the two m^7^G-related gene clusters varied in their clinical characteristics. To assess the prognostic value of the m^7^G-related genes, we constructed a prognostic model with nine gene risk profiles identified using univariate, multivariate, and LASSO Cox regression analyses. Functional analysis showed that DEGs in the low- and high-risk groups were associated with immune-related pathways as well as hormone synthesis and metabolism. Immune cell infiltration, immune pathways, and immune checkpoints were further compared between the low- and high-risk groups.

m^7^G modifications contribute to pathogenic infectivity and are associated with various diseases (22, 26–28). m^7^G modification may also be an essential process in tumorigenesis (22). However, the functions and association of m^7^G-related genes with patient survival are unclear. In this study, we constructed a prognostic model with nine m^7^G-related genes (*KLK7*, *LCE3E*, *ADH1C*, *AREG*, *KLK6*, *NTS*, *ERLIN2*, *ZBED2*, and *MAPK4*) that predicted OS in patients with LUSC. KLK6 and KLK7 have been reported as markers of gastric, breast, and colon cancers and may be associated with m^7^G modifications (29–31). Metzger et al. (32) reported that *LCE3E* is associated with poor survival in laryngeal squamous cell carcinoma, which corroborates our findings. Wang et al. (33) reported that ADH1C affects the prognosis and biological behavior of lung cancer; *ADH1C* scores were relatively high in the prognostic model. Yuan et al. (34) reported that TAZ (phospholipid-lysophospholipid transacylase) increased *EGFR* wild-type NSCLC sensitivity to gefitinib via AREG. NTS was also associated with lung cancer drug resistance proteins, which is consistent with our drug sensitivity results and also highlights the potential importance of m^7^G-related genes in determining drug sensitivity (35). In a recent report, ERLIN2 was suggested to be associated with METTL8 and CD8^+^ T-cell immune infiltration in LUSC (36), which corroborates our immune cell infiltration results; however, ERLIN2 was not associated with m^7^G modification.

m^6^A modification can affect CD8^+^ T-cell immune infiltration, while, in this study, infiltration of “T helper cells” was characterized by significant variation and high expression (37). This might be because m^7^G modification directly or indirectly affects CD8^+^ T cells under the influence of m^6^A modification in the immune response, thereby achieving and enhancing this effect (38). Immune infiltration could also be enhanced by the modification of tRNAs by m^7^G, which increases mRNA expression (39). m^7^G modification is considered to achieve similar effects as m^6^A modification and also assist m^6^A modification. For example, the cancer markers METTL8 and METTL1 may be modified by both m^6^A and m^7^G (40). In LUSC and adenocarcinoma, ZBED2 mainly regulates keratinization, which may be closely related to the pathological process of LUSC (41, 42). MAPK4 plays a major role in a variety of tumors, mainly involving immune-related pathways (43). m^7^G and Ψ modifications occur widely and are synergistically involved with m^6^A modifications in various intracellular processes. Although there are many reports on Ψ and m^6^A modifications in tumors, the role of m^7^G modifications has received little attention (40, 44). m^7^G modifications may be involved in multiple modes of cell death (45). Cell death often occurs at a large scale and requires the involvement of several factors in both apoptosis and cell scorch death mediated by immunity (46). This complex and sophisticated process is likely triggered by m^7^G modification alone or m^7^G assisting m6A modification (37). However, owing to lack of data, it is difficult to confirm whether these modifications cooperate in LUSC and thus requires further investigation.

In the analysis of DEGs between different risk groups, we found that they were mainly involved in the immune response and hormone metabolic effects, suggesting that m^7^G modifications may affect the prognosis of LUSC through immune-related functions as well as hormone regulation, although this requires further validation. Key antitumor infiltration immune-related functions were significantly expressed, suggesting that the immune function of patients with high-risk LUSC is altered, which is associated with m^7^G-related genes. In the drug sensitivity analysis, 6 of the 9 prognosis-related m^7^G-related genes were matched with 79 drugs, suggesting that m^7^G-related genes are also valuable for studying drug sensitivity.

Although we integrated information from multiple databases, this study still has limitations. First, we considered a large amount of microarray and sequencing data based on tumor tissue analysis. Therefore, our analyses may be affected by systematic bias. To overcome this problem, future studies with higher resolutions are needed. Second, the present study only performed bioinformatics analysis of m^7^G-related gene expression and patient survival in different databases, without in vivo or in vitro experiments. Further observational and empirical studies of m^7^G at the cellular and molecular levels could help to elucidate its biological role. Third, we found that m^7^G-related DEGs were mainly involved in the immune response and hormone metabolic effects; however, the relationship between immune-related functions and hormone regulation remains unclear.

In conclusion, based on the differential expression of m^7^G modification-associated genes between normal and LUSC tissues and the fact that our m^7^G-related gene risk profiles performed well as independent risk factors for predicting OS, we conclude that m^7^G modification is closely associated with the development of LUSC. Considering the association of DEGs in low- and high-risk groups with immunity and drug sensitivity, this study provides a new genetic marker for predicting the prognosis of patients with LUSC and provides an essential theoretical basis for future studies on the relationship between m^7^G modification-related genes and immunity and drug sensitivity in LUSC.

## DATA AVAILABILITY

Data will be made available upon reasonable request.

## SUPPLEMENTAL DATA

10.6084/m9.figshare.21757154.v2 Supplemental Tables S1–S4: https://doi.org/10.6084/m9.figshare.21757154.v2.

## GRANTS

This work was supported by National Natural Science Foundation of China Grant U21A20334.

## DISCLOSURES

No conflicts of interest, financial or otherwise, are declared by the authors. 

## AUTHOR CONTRIBUTIONS

Y.W. and S.Y. conceived and designed research; Y.W., Y.L., R.W., F.C., Y.G., Y.C., B.A., and S.Q. performed experiments; Y.W., Y.L., R.W., F.C., Y.G., Y.C., B.A., and S.Q. analyzed data; Y.W. interpreted results of experiments; Y.W. prepared figures; Y.W. drafted manuscript; Y.W. edited and revised manuscript; Y.W. and S.Y. approved final version of manuscript. 
